# Highly Efficient Graphene Oxide/Zinc Oxide/Lignin Catalyst for Photocatalytic Degradation of Methylene Blue and Gentian Violet

**DOI:** 10.3390/nano15171342

**Published:** 2025-09-01

**Authors:** Tamanna Yakub, Anupama Asthana, Sunita Sanwaria, Ajaya Kumar Singh, Sónia A. C. Carabineiro

**Affiliations:** 1 Department of Chemistry, Govt. V.Y.T. Post Graduate Autonomous College Durg, Durg 491001, India; tamannayakub10@gmail.com; 2 Department of Chemistry, Government Nagarjuna Post Graduate College of Science, Raipur 492010, India; sanwaria.sunita@gmail.com; 3 School of Chemistry & Physics, University of KwaZulu–Natal, Durban 3630, South Africa; 4 LAQV-REQUIMTE, Department of Chemistry, NOVA School of Science and Technology, Universidade NOVA de Lisboa, 2829-516 Caparica, Portugal; sonia.carabineiro@fct.unl.pt

**Keywords:** cationic dyes, photocatalytic degradation, lignin, ZnO nanoparticles, graphene oxide, nanocomposite, methylene blue, gentian violet

## Abstract

This study presents a comprehensive investigation of a novel graphene oxide/zinc oxide/lignin (GO/ZnO/lignin) nanocomposite for the photocatalytic degradation of methylene blue (MB) and gentian violet (also known as crystal violet, CV) dyes in aqueous solutions. The nanocomposite was synthesized through a hydrothermal method and thoroughly characterized using Fourier transform infrared spectroscopy (FTIR), X-ray diffraction (XRD), scanning electron microscopy (SEM), and energy-dispersive X-ray spectroscopy (EDX). FTIR spectra confirmed the successful incorporation of functional groups from all components, while XRD patterns revealed a well-crystallized structure with characteristic peaks. SEM micrographs showed a uniform, hierarchical morphology and EDX analysis verified the elemental composition and distribution. Under ultraviolet (UV) irradiation, the nanocomposite exhibited remarkable photocatalytic degradation efficiency (~97%) for both MB and CV. Key operational parameters were systematically evaluated, including pH (2–10), catalyst dosage (0.005–0.04 g/20 mL), and initial dye concentration (10–20 ppm). Optimal performance was achieved at pH 10, with a catalyst dosage of 0.03–0.04 g/20 mL and lower dye concentrations. The enhanced photocatalytic activity can be attributed to the synergistic effects coming from GO’s electron transport capabilities, ZnO’s strong photocatalytic activity and lignin’s additional degradation sites. Furthermore, the nanocomposite demonstrated excellent reusability, retaining nearly 60% of its degradation capacity after four cycles, outperforming its individual components. These results highlight the potential of this composite material for sustainable wastewater treatment applications.

## 1. Introduction

Water pollution coming from industrial effluents, particularly those containing persistent organic pollutants, such as synthetic dyes, represents a significant environmental challenge [1]. Cationic dyes, which are widely used in the textile, paper, and other industries, are especially problematic due to their complex molecular structures and resistance to conventional treatment methods [2]. This persistence in aqueous environments not only contaminates water sources but also poses serious toxicological risks to living organisms [3]. Traditional water treatment methods have proven inadequate for fully removing these persistent pollutants [4], highlighting the need for more effective and sustainable treatment technologies [5]. Advanced oxidation processes (AOPs), particularly photocatalytic degradation, have emerged as promising solutions for eliminating these recalcitrant organic pollutants [6].

The rapid industrialization and expansion of textile manufacturing have raised growing concerns over the discharge of synthetic dyes into water bodies, posing a significant environmental challenge. Among these pollutants, methylene blue (MB) and gentian violet, also known crystal violet (CV), are widely used organic dyes known for their high stability and resistance to conventional treatment methods, making their removal from wastewater a critical environmental priority. These dyes not only degrade water quality but also pose serious risks to aquatic ecosystems and human health due to their toxic [7], carcinogenic [8], and mutagenic properties.

Photocatalysis has emerged as a promising advanced oxidation process for degrading organic pollutants, offering an environmentally friendly and sustainable approach to water treatment. In recent years, zinc oxide (ZnO) [9] has attracted significant attention as a photocatalyst due to its wide band gap [10], strong oxidizing ability, low cost, and environmental compatibility [11]. However, its practical application is limited by a high electron–hole recombination rate and limited visible-light absorption, highlighting the need for the development of modified ZnO-based composites with enhanced photocatalytic performance [12].

The integration of graphene oxide (GO) with ZnO has demonstrated significant potential for improving photocatalytic efficiency by enhancing charge separation and increasing the surface area available for catalytic reactions [13,14]. GO’s unique two-dimensional structure and excellent electron transport properties make it an ideal support material for semiconductor photocatalysts [15].

Moreover, the incorporation of lignin, a natural and abundant biopolymer rich in functional groups [16], into the GO/ZnO composite offers an innovative strategy to further enhance photocatalytic performance by improving light absorption and promoting the degradation of pollutants [17]. Lignin, the second most abundant natural polymer after cellulose, is a complex, three-dimensional, amorphous biopolymer composed of phenylpropane units interconnected by various chemical bonds [18], including β-O-4, α-O-4, and C–C linkages. Its unique structural features, particularly the abundance of functional groups, such as hydroxyl, methoxyl, and carbonyl moieties, make it an attractive candidate for environmental remediation and catalytic applications [19]. As a renewable and biodegradable by-product predominantly derived from the pulp and paper industry, lignin offers significant sustainability and cost advantages [20]. The aromatic rings and oxygen-containing functional groups in its structure enable strong interactions with both organic pollutants and metal oxide surfaces [21], making lignin especially valuable in the development of advanced composite materials for photocatalytic applications [22]. Furthermore, lignin’s potential to act as a natural sensitizer, enhancing light absorption and facilitating electron transfer processes, has fostered growing interest in its integration into photocatalytic systems.

This paper presents the development of a highly efficient GO/ZnO/lignin composite catalyst, representing a significant advancement in photocatalytic technology. The synergistic interaction among GO, ZnO, and lignin effectively addresses the limitations of conventional photocatalysts, offering a sustainable and enhanced solution for the degradation of persistent organic pollutants, such as methylene blue (MB) and crystal violet (CV) [23]. This novel composite combines the unique advantages of each component: GO’s exceptional electronic conductivity and charge transport capabilities [24], ZnO’s strong intrinsic photocatalytic activity, and lignin’s environmental sustainability and functional versatility. Together, these properties enable a more effective and eco-friendly approach to wastewater treatment.

## 2. Experimental Procedure

### 2.1. Chemical Reagents

Zinc sulfate heptahydrate (98%) (ZnSO_4_·7H_2_O), sodium hydroxide (NaOH), ammonium hydroxide (NH_4_OH), graphite powder, hydrochloric acid (HCl), sodium nitrate (NaNO_3_), sulfuric acid (98%) (H_2_SO_4_), potassium permanganate (KMnO_4_), formaldehyde (HCHO), hydrogen peroxide (H_2_O_2_), lignin, methylene blue, and crystal violet were all purchased from Sigma-Aldrich (St. Louis, MO, USA). All reagents and solvents were of commercial grade and used without further purification. Deionized distilled water was used as the solvent throughout the experiments.

### 2.2. Instrumentation

FTIR spectra were recorded using a Thermo Nicolet Avtar 370 spectrophotometer ((Thermo Fisher Scientific, Waltham, MA, USA) equipped with a deuterated triglycine sulfate (DTGS) detector, operating at a resolution of 4 cm^−1^ with KBr disc sample preparation. XRD analysis was performed on a Bruker D8 Advance diffractometer (Bruker, Ettlingen, Germany) using Ni-filtered Cu-Kα radiation (λ = 1.5405 Å), with scans conducted over a 2θ range of 2° to 80° at a step size of 0.02°. Surface morphology was examined using a Carl Zeiss ultra-high-resolution field emission scanning electron microscope (UHR FESEM) Gemini SEM 500 (Carl Zeiss, Jena, Germany) with a resolution of 0.8 nm and a probe current range of 3 pA to 100 nA. Elemental composition was analyzed by EDX, equipped with a silicon drift detector (SDD) for high-resolution elemental mapping. Ultraviolet–visible (UV–vis) spectra were obtained on a Shimadzu UV-1900i spectrophotometer (Shimadzu, Kyoto, Japan), operating over a wavelength range of 190–1100 nm, with 1 nm resolution and high-speed scanning. Collectively, these analytical techniques provided comprehensive insights into the nanocomposite’s morphology, surface characteristics, and structural properties. Electrochemical measurements were performed using a CHI660E electrochemical workstation (CH Instruments, Bee Cave, TX, USA) in a standard three-electrode configuration. The working electrode was prepared by coating the synthesized materials on FTO glass substrates, with Pt wire as the counter electrode and Ag/AgCl as the reference electrode. Transient photocurrent responses were measured under chopped light illumination (λ > 420 nm) in 0.1 M Na_2_SO_4_ electrolyte solution.

### 2.3. Synthesis of Nanocomposite Materials

#### 2.3.1. Synthesis of Graphene Oxide (GO)

Graphene oxide (GO) was synthesized using a modified Hummers’ method. Briefly, 1.00 g of graphite and 0.5 g of sodium nitrate (NaNO_3_) were mixed with 23 mL of 98% sulfuric acid (H_2_SO_4_) under continuous stirring at 66 °C. The mixture was then allowed to cool and rest at room temperature for 30 min. Subsequently, 3.00 g of potassium permanganate (KMnO_4_) was added gradually while maintaining the reaction in an ice bath to control the exothermic nature of the process. The resulting suspension was sonicated for 1 h to ensure uniform oxidation. The mixture was then heated to 98 °C, followed by the careful addition of 50 mL of distilled water, producing a brown suspension. This suspension was further diluted with 700 mL of warm distilled water, and 12 mL of hydrogen peroxide (H_2_O_2_) was added. The color changed from brown to yellow, indicating the reduction of residual permanganate species. Finally, the final mixture was thoroughly washed with distilled water until neutral pH and then dried at 60 °C for 24 h to obtain the graphene oxide powder.

#### 2.3.2. Synthesis of ZnO Nanoparticles

ZnO nanoparticles were synthesized by a simple chemical precipitation method using zinc nitrate hexahydrate as the precursor and sodium hydroxide as the precipitating agent. A sodium hydroxide solution was added dropwise to the zinc nitrate solution under vigorous stirring at room temperature, maintaining the pH at approximately 12. The resulting white precipitate was allowed to age for several hours to facilitate crystal growth, then collected by centrifugation, thoroughly washed multiple times with deionized water and ethanol to remove impurities, and dried at 60 °C. Finally, the dried material was calcined at 400 °C for 2 h to obtain highly crystalline ZnO nanoparticles.

#### 2.3.3. Synthesis of GO/ZnO Composite

The GO/ZnO composite was prepared via a facile solution mixing method. Initially, the previously synthesized GO was ultrasonically dispersed in deionized water to achieve thorough exfoliation and stable suspension. Subsequently, ZnO nanoparticles were added dropwise to the GO suspension under continuous stirring. The mixture was stirred for an extended period to ensure uniform distribution of ZnO nanoparticles onto the GO sheets, facilitated by electrostatic interactions and the presence of oxygen-containing functional groups on GO. The resulting suspension was then centrifuged, washed thoroughly with deionized water to remove unbound particles, and dried to obtain the GO/ZnO composite powder.

#### 2.3.4. Synthesis of GO/ZnO/Lignin Nanocomposite

A novel GO/ZnO/lignin nanocomposite was synthesized using a one-step hydrothermal method. First, 0.8 g of the GO/ZnO nanocomposite was dispersed in 50 mL of distilled water. Then, 0.8 g of lignin was added to the suspension. The mixture was subjected to ultrasonication for 30 min to ensure homogeneous dispersion, followed by continuous stirring for 48 h to promote effective integration of lignin into the composite matrix. The resulting product was then centrifuged, washed thoroughly with ethanol to remove unreacted components, and freeze-dried to obtain the GO/ZnO/lignin nanocomposite.

### 2.4. Photocatalytic Performance Evaluation

#### 2.4.1. Dark Adsorption Test

Prior to conducting photocatalytic experiments, dark adsorption tests were performed to evaluate the adsorption capacity of the materials and eliminate any influence of adsorption on the photodegradation performance. In a typical dark adsorption experiment, 50 mg of the photocatalyst was dispersed in 100 mL of dye solution (either MB or CV) with an initial concentration of 10 mg/L. The suspension was stirred in complete darkness for 60 min to establish adsorption–desorption equilibrium. Samples were collected at regular intervals (10, 20, 30, 45, and 60 min) and analyzed using UV–Vis spectroscopy to monitor dye concentration. Adsorption equilibrium was considered achieved when no significant change in dye concentration was observed between consecutive measurements.

#### 2.4.2. Individual Component Performance Testing

To evaluate the individual contributions of each component, the photocatalytic performance of pure GO, ZnO nanoparticles, and lignin was assessed under identical experimental conditions. For each test, 50 mg of the respective material was dispersed in 100 mL of dye solution (10 mg/L of either MB or CV), and the photocatalytic degradation was carried out following the same protocol used for the composite materials. Additionally, the adsorption performance of the GO/ZnO/lignin composite was systematically investigated under dark conditions using dye solutions of varying concentrations (5, 10, 15, 20, and 25 mg/L). These experiments aimed to determine the composite’s maximum adsorption capacity and to establish adsorption isotherms for a comprehensive understanding of its adsorption behavior.

#### 2.4.3. Photocatalytic Experimental Setup

Photocatalytic experiments were carried out in a custom-built photoreactor equipped with a 300 W xenon lamp (λ > 420 nm) serving as the visible light source, positioned 15 cm above the reaction vessel. The light intensity at the reaction surface was measured to be 100 mW/cm^2^ using a calibrated power meter. In a typical experiment, 50 mg of photocatalyst was dispersed in 100 mL of dye solution (initial concentration: 10 mg/L), maintaining a photocatalyst-to-solution ratio of 0.5 g/L. The suspension was initially stirred in the dark for 30 min to establish adsorption–desorption equilibrium, ensuring that any subsequent changes in dye concentration were attributable solely to photocatalytic activity.

During visible light irradiation, the suspension was continuously stirred to maintain homogeneous dispersion and prevent catalyst sedimentation. At predetermined time intervals (0, 15, 30, 60, 90, and 120 min), 3 mL aliquots were withdrawn and immediately centrifuged to separate the photocatalyst, and the supernatant was analyzed using UV–Vis spectroscopy to monitor the degradation kinetics. The photocatalytic efficiency was calculated using the following equation:

Efficiency (%) = (C_0_′ − C)/C_0_′ × 100,

where C_0_′ is the equilibrium dye concentration after 30 min dark adsorption and C is the concentration at time t during photocatalytic treatment.

## 3. Results and Discussion

### 3.1. Characterization Results

#### 3.1.1. FTIR

**Figure 1:**
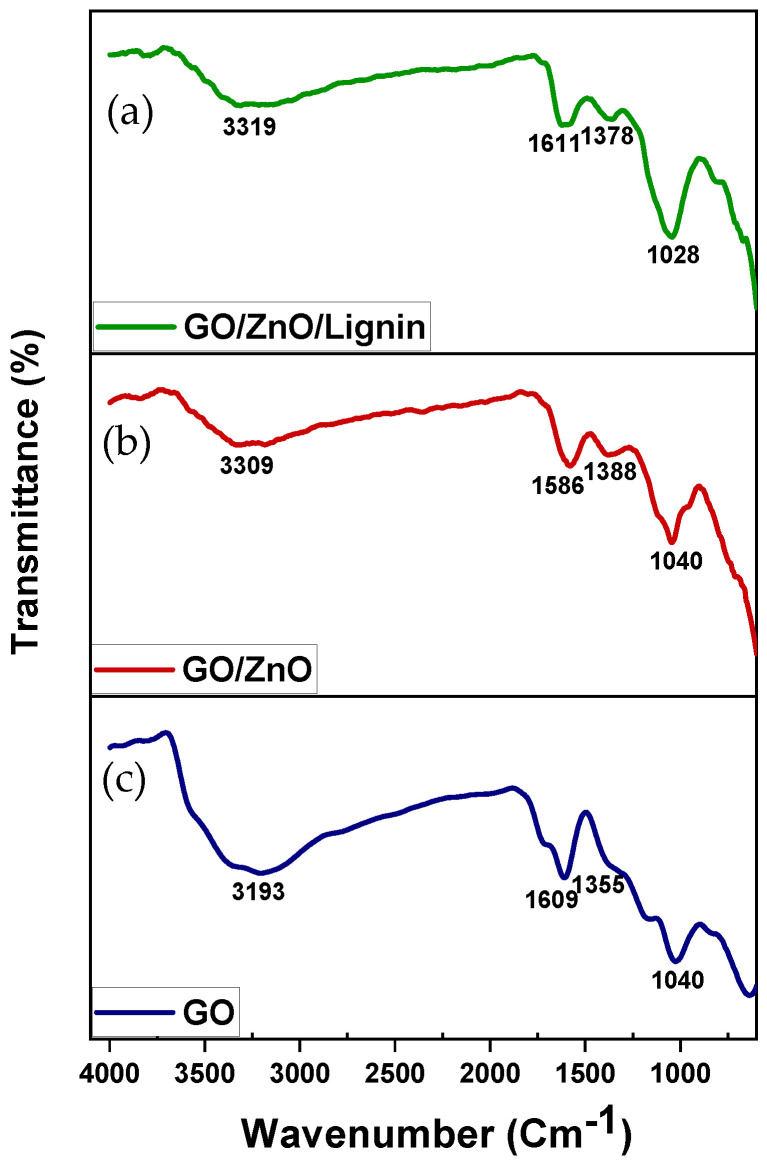
FTIR spectra of (**a**) GO/ZnO/lignin nanocomposite; (**b**) GO/ZnO nanoparticles; and (**c**) GO.

The FTIR spectra of GO (Figure 1, bottom), GO/ZnO (Figure 1, middle), and GO/ZnO/lignin nanocomposite (Figure 1, top) confirm the successful formation of the composite. GO shows characteristic peaks at 3130 cm^−1^ (O–H stretching), 1609 cm^−1^ (C=C aromatic stretching), and 1040 cm^−1^ (C–O stretching). The GO/ZnO spectrum exhibits peaks at 3309 cm^−1^ (O–H stretching), 1581 cm^−1^ (C=C stretching), and 1040 cm^−1^, attributed to C–O and Zn–O vibrations. The GO/ZnO/lignin nanocomposite displays peaks at 3319 cm^−1^ (O–H stretching from lignin and GO), 1611 and 1373 cm^−1^ (C=C aromatic stretching and C–H bending), and 1028 cm^−1^ (C–O stretching and Zn–O vibrations). Shifts in peak positions and changes in intensity across spectra indicate strong interactions among GO, ZnO, and lignin, confirming successful synthesis of the nanocomposite and effective integration of its components.

#### 3.1.2. XRD

**Figure 2:**
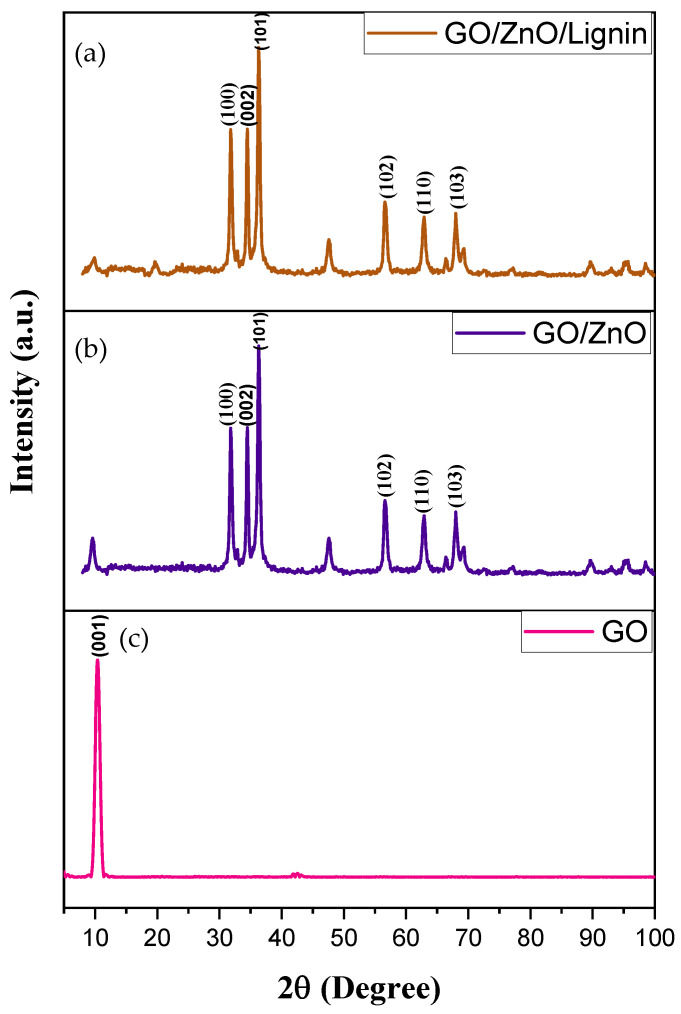
XRD spectra of (**a**) GO/ZnO/lignin nanocomposite, (**b**) GO/ZnO nanoparticles, and (**c**) graphene oxide (GO).

Figure 2 presents the XRD patterns of ZnO nanoparticles, GO, and the GO/ZnO/lignin nanocomposite. The crystallite size of the nanomaterials was calculated using the Debye–Scherrer formula [25]:

(1) $$\frac{1}{d_{hkl}^{2}}=\frac{4}{3}\left[\frac{h^{2}+hk+\;k^{2}}{a^{2}}\right]+\;\frac{l^{2}}{c^{2}}$$

where the following apply:

d = interplanar spacing.
h, k, l = Miller indices of the crystal plane.
‘a’ and ‘c’ = lattice constants, with values a = 3.2554 Å, c = 4.9569 Å, and c/a = 1.5227 Å.

The finite broadening observed in the XRD diffraction peaks indicates that the synthesized materials are within the nanometer size range. The average crystallite sizes of ZnO, GO, and lignin-based nanocomposite were calculated using the Debye–Scherrer equation [26].

(2) $$D=\frac{0.9\;\lambda }{\beta \;cos\;cos\;\theta \;}$$

where the following apply:

λ = X-ray wavelength (Cu Kα radiation) = 0.15406 nm (or 1.5406 Å).
β = full width at half maximum (FWHM) of the XRD peak (in radians).
θ = Bragg diffraction angle (in radians).

The X-ray diffraction patterns in Figure 2 reveal the distinct structural characteristics of the GO, GO/ZnO nanoparticles, and GO/ZnO/lignin nanocomposite. Pure GO exhibits a characteristic sharp peak at 10° (2θ) corresponding to the (001) reflection (JCPDS 87-1526), indicating a well-ordered layered structure with expanded interlayer spacing of 8.4 Å due to oxygen-containing functional groups. The GO/ZnO composite displays both the GO peak and multiple ZnO reflections at 31.8°, 34.5°, 36.3°, 47.6°, 56.6°, and 62.9°, corresponding to (100), (002), (101), (102), (110), and (103) planes of the hexagonal wurtzite ZnO (JCPDS 36-1451). The most intense peak, at 36.3° represents the (101) plane, confirming successful ZnO crystallization within the GO matrix.

The GO/ZnO/lignin nanocomposite maintains similar peak positions with slight intensity modifications, indicating successful lignin incorporation without disrupting the crystalline phases. Using the Scherrer equation applied to the most intense (101) peak, crystallite sizes were calculated as approximately 20 nm for GO/ZnO and 18 nm for GO/ZnO/lignin composite. The slight size reduction suggests lignin acts as a growth inhibitor during ZnO crystallization.

Table 1 presents the comprehensive XRD analysis of the synthesized GO/ZnO/lignin nanocomposites, displaying the crystallographic data for zinc oxide phases identified in the diffraction patterns. The table systematically lists seven distinct diffraction peaks observed at 2θ values of 31.58°, 34.48°, 36.32°, 47.65°, 56.67°, 62.88°, and 68.02°, corresponding to the Miller indices of the (100), (002), (101), (102), (110), (103), and (201) crystallographic planes of the hexagonal wurtzite ZnO structure, respectively. The calculated d-spacing values range from 2.81 Å for the (100) plane to 1.38 Å for the (201) plane, which are consistent with the JCPDS card 36-1451 for ZnO. The most prominent reflection at 36.32° corresponds to the (101) plane with a d-spacing of 2.47 Å, representing the characteristic peak used for crystallite size calculations. The systematic decrease in d-spacing values with increasing 2θ angles follows the expected crystallographic relationship, confirming the successful formation of well-crystallized ZnO nanoparticles within the GO/lignin matrix and validating the phase purity of the synthesized nanocomposite material.

#### 3.1.3. SEM

**Figure 3:**
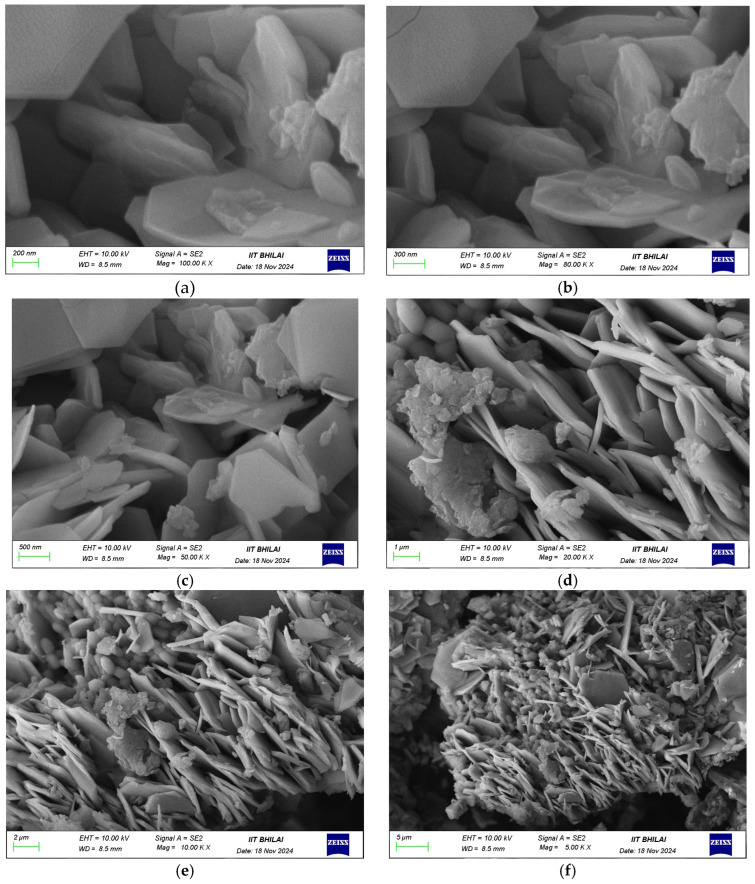
SEM images of GO/ZnO/lignin nanocomposite: (**a**) 200 nm scale showing flower-like structures; (**b**) 200 nm scale detailed view; (**c**) 5 μm scale showing network structure; (**d**) 5 μm scale overview; (**e**) 2 μm scale showing integration; (**f**) 2 μm scale detailed morphology.

The SEM micrographs illustrate the hierarchical morphology of the GO/ZnO/lignin nanocomposite at different magnifications. At the 200 nm scale (Figure 3, top row), flower-like ZnO nanostructures with petal-like protrusions are clearly observed, indicating successful ZnO growth on the wrinkled GO sheets. At a 5 μm scale (Figure 3, middle row), the images reveal an interconnected network in which ZnO particles are uniformly dispersed across the GO substrate, with lignin serving as a binder that enhances surface roughness. At a 2 μm scale (Figure 3, bottom row), the intimate integration of the components becomes more apparent, as ZnO particles are well-anchored onto the GO sheets through lignin-mediated interactions, resulting in a structurally stable composite. The surface exhibits notable roughness and porosity, which are advantageous for applications requiring high surface area and enhanced mass transfer. The homogeneous distribution of ZnO and lignin without significant agglomeration reflects the effectiveness of the synthesis and the strong interactions among GO, ZnO, and lignin, contributing to the composite’s structural stability and functional performance.

Additional SEM images of the individual components are presented in the Supporting Information for comparison. Figure S1 shows the characteristic wrinkled sheet morphology of GO; Figure S2 displays the well-defined crystalline shapes of ZnO nanoparticles; and Figure S3 illustrates the complex, amorphous structure of lignin. Further details can be found in the Supporting Information.

#### 3.1.4. EDX

The EDX spectrum and elemental analysis of the GO/ZnO/lignin composite confirm its chemical composition and elemental distribution. The spectrum displays three dominant peaks corresponding to carbon (C), oxygen (O), and zinc (Zn), verifying successful incorporation of all composite constituents. The quantitative analysis indicates carbon as the most abundant element, with a weight percentage of 51.3% and an atomic percentage 67.1%, primarily derived from the carbon frameworks of GO and lignin. Oxygen is the second most abundant, with 28.6 wt% (28.1 at%), attributed to the oxygen-containing functional groups in GO and lignin, as well as ZnO.

**Figure 4:**
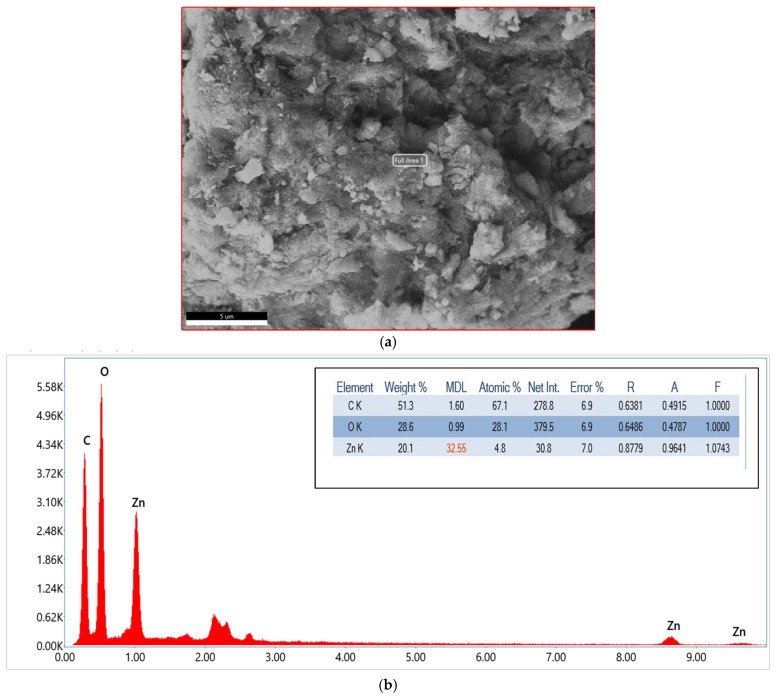
(**a**) SEM image of GO/ZnO/lignin nanocomposite used for EDX analysis; (**b**) EDX spectrum of GO/ZnO/lignin nanocomposite.

Zinc accounts for 20.1 wt% (4.8 at%), confirming the effective incorporation of ZnO nanoparticles into the matrix. The reported error margins (~6.9–7.0%) reflect standard measurement uncertainties, while the R, A, and F values represent correction factors applied during the EDX analysis. The corresponding SEM images (Figure 4) reveal a heterogeneous surface morphology characterized by aggregated particles and layered structures, in agreement with the elemental mapping. These findings confirm the formation of a well-integrated nanocomposite, where GO provides a carbon-rich scaffold, ZnO contributes with active photocatalytic sites, and lignin enhances both structural and compositional balance. This synergy results in a nanocomposite with optimized stoichiometry morphology, well-suited for photocatalytic applications.

### 3.2. Photocatalytic Degradation Kinetics

#### 3.2.1. Degradation of MB by GO/ZnO/Lignin Nanocomposite

**Figure 5:**
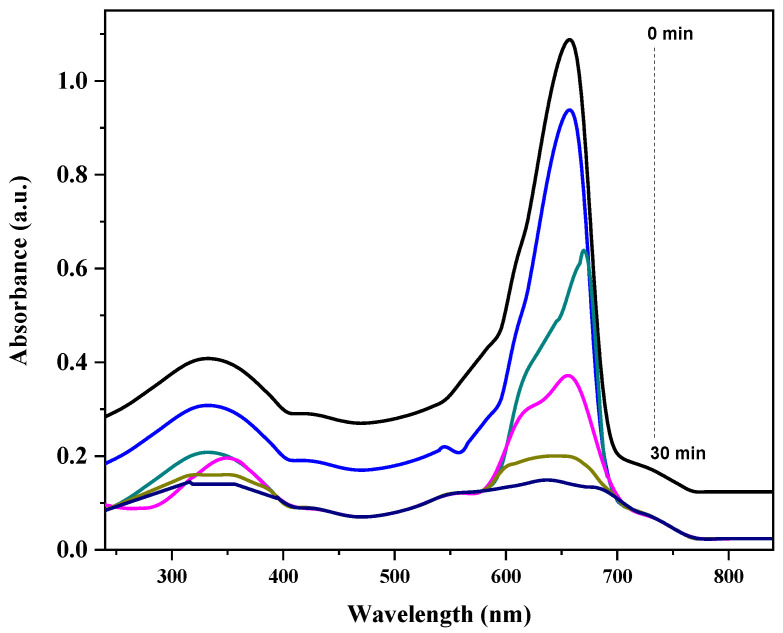
Degradation kinetics of MB Dye by GO/ZnO/lignin nanocomposite.

The UV–vis absorption spectra demonstrate the photocatalytic degradation of MB by the GO/ZnO/lignin nanocomposite under UV irradiation over a 30 min period. The characteristic absorption peak of MB, located between 600 and 700 nm and associated with n→π* transitions in the dye’s chromophoric structure, gradually decreases in intensity from the initial measurement (0 min) to the final one (30 min). This decline in peak height indicates the progressive disruption of MB’s conjugated system, confirming effective photocatalytic degradation. At 0 min, the absorption is highest, reflecting the initial dye concentration, but it steadily diminishes as the reaction proceeds. A slight blue shift in the peak position suggests the generation of intermediate degradation products during the process. The consistent reduction in absorption across the visible spectrum (400–800 nm) further supports the nanocomposite’s efficiency in oxidizing and decomposing MB molecules. This enhanced performance results from the synergistic interaction of GO’s excellent electron mobility, ZnO’s photocatalytic activity and lignin’s adsorption and light-harvesting capabilities. The near-complete disappearance of the absorption peak after 30 min confirms the high photocatalytic efficiency of the GO/ZnO/lignin nanocomposite under UV irradiation (Figure 5).

#### 3.2.2. Degradation of CV by GO/ZnO/Lignin Nanocomposite

**Figure 6:**
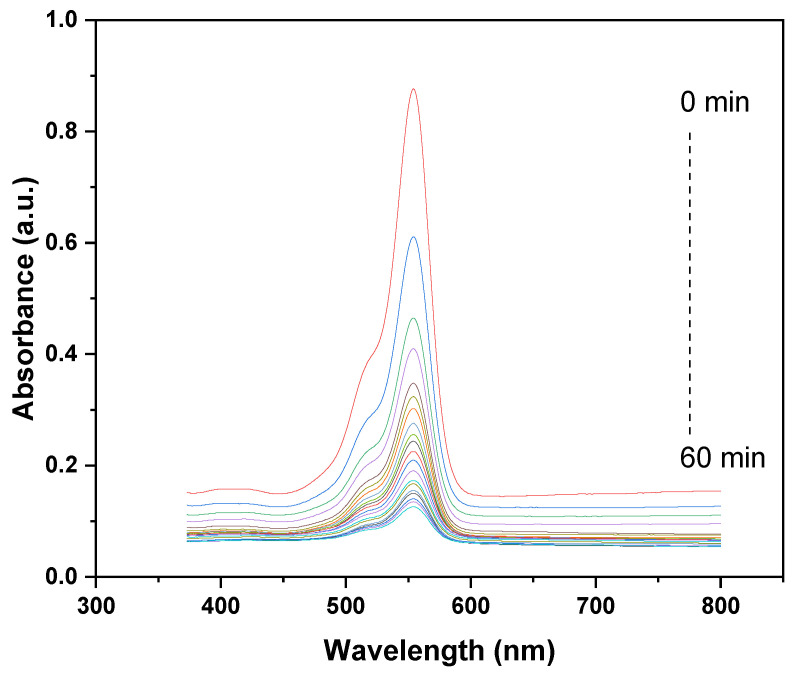
Degradation kinetics of CV dye by GO/ZnO/lignin nanocomposite.

The UV–vis absorption spectra illustrate the photocatalytic degradation kinetics of CV using the GO/ZnO/lignin nanocomposite over a 60 min UV irradiation period. A prominent absorption peak appears around 550–600 nm, characteristic of CV’s conjugated chromophoric structure. The gradual decrease in peak intensity, from the initial measurement at 0 min (highest peak) to the final one at 60 min (lowest peak), indicates progressive photodegradation of CV. The sharp decline during the first 30 min suggests rapid initial degradation, followed by a slower rate in the latter half, which is typical of photocatalytic degradation behavior. This trend highlights the effective photocatalytic activity of the GO/ZnO/lignin nanocomposite, where the synergistic interplay between GO’s efficient electron transport, ZnO’s photocatalytic properties, and lignin’s adsorptive capacity promotes the efficient breakdown of CV molecules. The near-complete disappearance of the absorption peak after 60 min confirms the high photocatalytic efficiency of the nanocomposite under the tested conditions (Figure 6).

**Figure 7:**
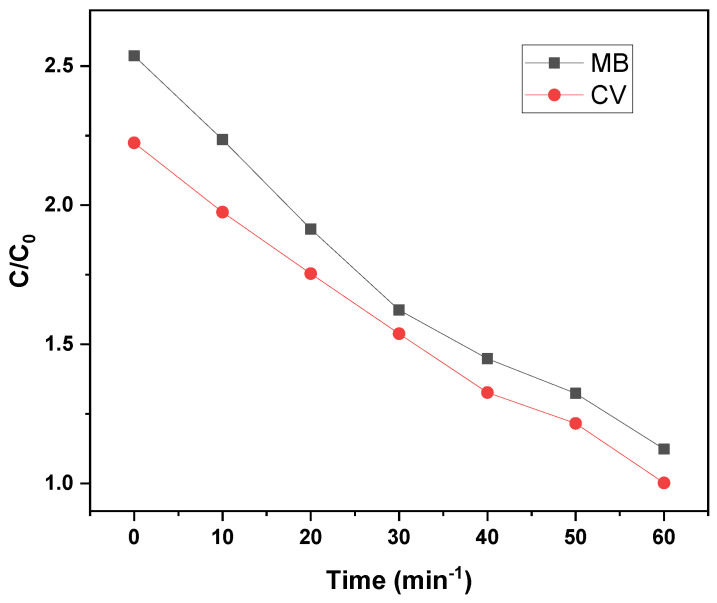
Effect of GO/ZnO/lignin nanocomposite concentration on degradation of MB and CV dyes over time.

Figure 7 illustrates the concentration decay profiles (C/C_0_) of MB and CV dyes during photocatalytic degradation over 30 min and 60 min, respectively. MB exhibits a slightly faster initial degradation, beginning from a higher relative concentration (C/C_0_ ≈ 2.5) compared to CV (C/C_0_ ≈ 2.2). Both dyes exhibit a similar decreasing trend, with C/C_0_ values approaching approximately 1.0–1.2 by the end of their respective irradiation periods, confirming effective photocatalytic removal. The observed decline in dye concentration follows a pseudo-first-order kinetic model, consistent with the Langmuir–Hinshelwood mechanism, suggesting that the degradation rate is governed by the adsorption of dye molecules onto the catalyst surface:

C_0_ = C_t_e^−kt^ (3)

**Figure 8:**
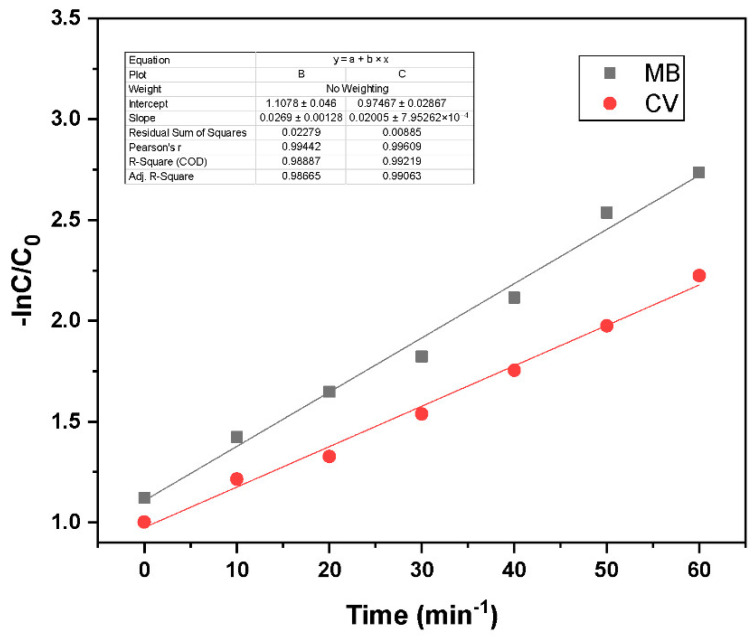
Langmuir–Hinshelwood first-order kinetic model fit for GO/ZnO/lignin nanocomposite at different reaction conditions (MB and CV concentration 15 ppm, catalyst loading 0.03 g).

−ln C_t_/C_0_ = ktm (4)

where C_0_ and C_t_ represent the initial and time-dependent dye concentrations, respectively, and k is the reaction rate constant (min^−1^). Figure 8 shows the Langmuir–Hinshelwood first-order kinetic model analysis, with plots of −ln(C/C_0_) versus time for both dyes. The observed linear trends confirm that the photodegradation of MB and CV follows first-order reaction kinetics. MB exhibits a steeper slope than CV, indicating a faster degradation rate under identical conditions. The experiments were performed with an initial dye concentration of 15 ppm and a photocatalyst loading of 0.03 g. The high coefficient of determination (R^2^) values validate the suitability of the Langmuir–Hinshelwood model in describing the photocatalytic degradation process.

#### 3.2.3. Effect of pH Degradation of GO/ZnO/Lignin Nanocomposite

**Figure 9:**
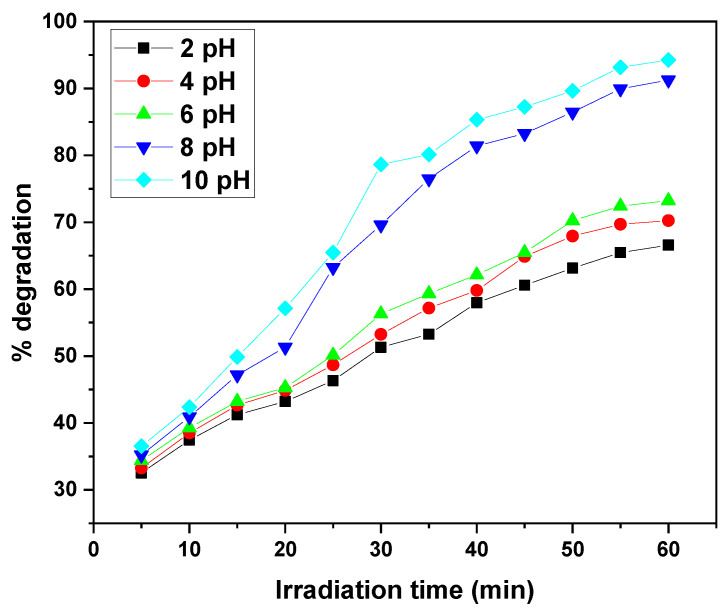
Effect of solution pH on removal efficiency of MB dye by GO/ZnO/lignin nanocomposite (initial concentration: 15 ppm, catalyst dosage: 0.03 g, temperature: 30 °C, time: 30 min).

**Figure 10:**
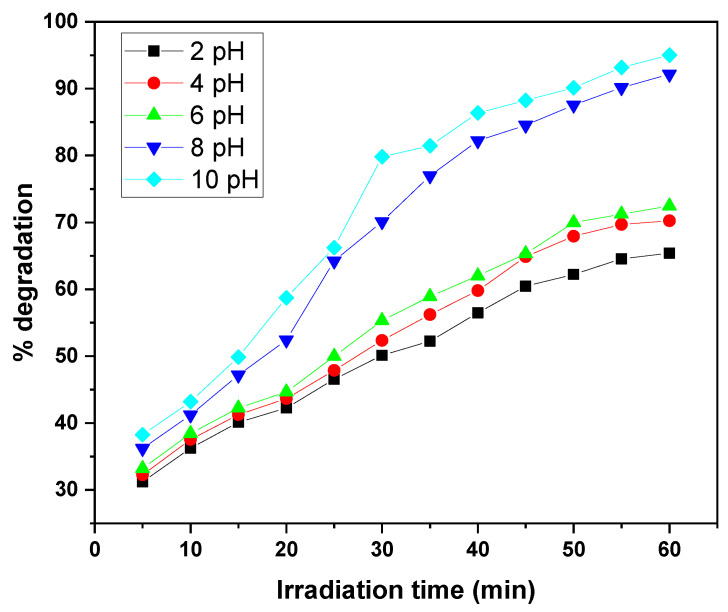
Effect of solution pH on removal efficiency of CV dye by GO/ZnO/lignin nanocomposite (initial concentration: 15 ppm, catalyst dosage: 0.03 g, temperature: 30 °C, time: 60 min).

The photocatalytic degradation of MB and CV by the GO/ZnO/lignin nanocomposite exhibits a clear dependence on solution pH under identical conditions (15 ppm dye concentration, 0.03 g catalyst, 30 °C, 30–60 min of UV irradiation). The degradation efficiency steadily increases from acidic to basic pH, with optimal performance observed at pH 10, achieving approximately 90–95% removal after 30–60 min. The lowest degradation efficiencies (~65%) occur at pH 2. This enhancement under alkaline conditions is attributed to the increased generation of hydroxyl radicals (•OH), which are the primary oxidizing species in the photocatalytic process. Intermediate pH values (4, 6, 8) show gradual improvements in efficiency, with pH 8 outperforming acidic conditions. This behavior is explained by the nanocomposite’s surface charge: at higher pH, the catalyst surface becomes negatively charged, enhancing electrostatic attraction with the positively charged dye molecules, thereby facilitating degradation. The similar pH-dependent trends observed for both MB and CV suggest that the nanocomposite’s surface properties, rather than the molecular structure of the dyes, primarily govern the photocatalytic behaviour (Figure 9 and Figure 10).

#### 3.2.4. Effect of Dose Degradation of GO/ZnO/Lignin Nanocomposite

**Figure 11:**
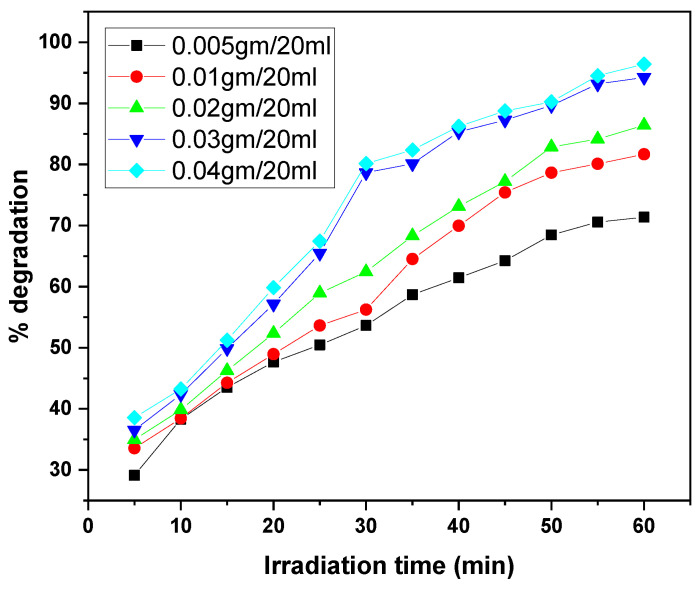
Effect of catalyst dosage on MB dye removal by GO/ZnO/lignin nanocomposite (initial concentration: 15 ppm, temperature: 30 °C, time: 30 min).

**Figure 12:**
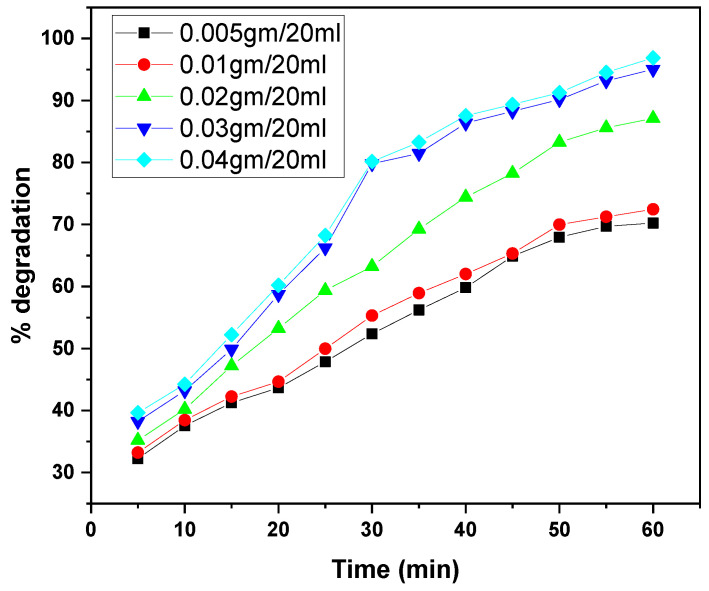
Effect of catalyst dosage on CV dye removal by GO/ZnO/lignin nanocomposite (initial concentration: 15 ppm, temperature: 30 °C, time: 60 min).

The graphs illustrate the photocatalytic degradation performance of the GO/ZnO/lignin nanocomposite at varying dosages (0.005 to 0.04 g per 20 mL) for the removal of two dyes, MB and CV, over irradiation times ranging from 30 to 60 min. In both cases, increasing the catalyst dosage enhanced degradation efficiency, with the highest dosages (0.03–0.04 g/20 mL) achieving approximately 95% degradation. This improvement is attributed to the increased number of active sites and the expanded photocatalytic surface area available at higher dosages. The degradation kinetics for both dyes exhibited a rapid initial phase followed by a gradual plateau as the active sites became saturated. MB showed slightly superior maximum degradation (~95%) compared to CV (~90%) under identical conditions, likely due to differences in molecular structure and dye–catalyst interactions. Overall, these results confirm the nanocomposite’s efficacy as a photocatalyst and highlight the importance of dosage optimization for effective dye removal in wastewater treatment (Figure 11 and Figure 12).

#### 3.2.5. Effect of Concentration Degradation of GO/ZnO/Lignin Nanocomposite

**Figure 13:**
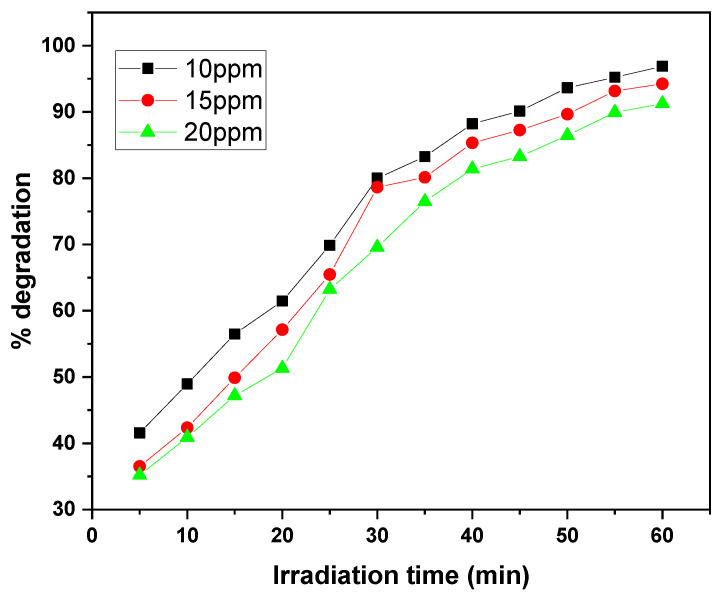
Effect of dye concentration on MB removal rate by GO/ZnO/lignin nanocomposite (catalyst dosage: 0.03 g, temperature: 30 °C, time: 30 min).

**Figure 14:**
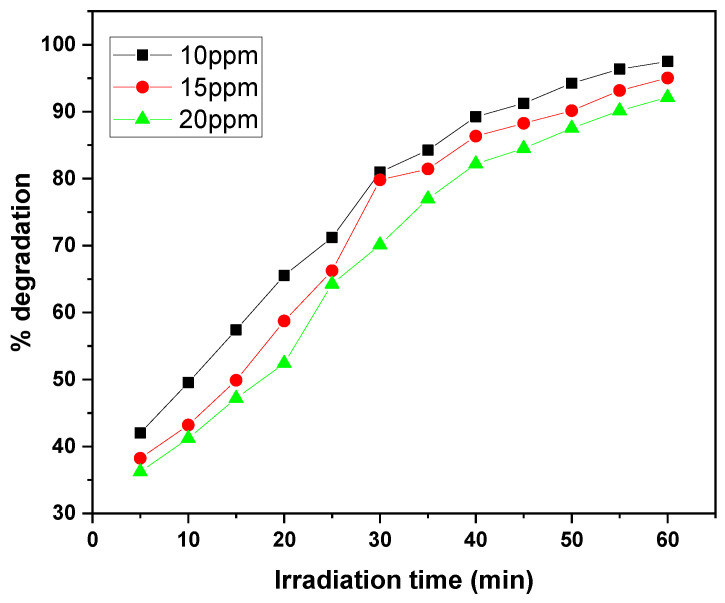
Effect of dye concentration on CV removal rate by GO/ZnO/lignin nanocomposite (catalyst dosage: 0.03 g, temperature: 30 °C, time: 60 min).

The graphs illustrate the photocatalytic degradation performance of the GO/ZnO/lignin nanocomposite at different initial dye concentrations (10, 15, and 20 ppm) for MB and CV over 30 to 60 min of irradiation. At the lowest concentration (10 ppm), both dyes exhibited the highest degradation efficiency, reaching approximately 97% removal within the 30 to 60 min irradiation period. As the initial concentration increased to 20 ppm, the degradation efficiency decreased to around 90%, with both dyes following a similar trend (Figure 13 and Figure 14).

This concentration-dependent behavior can be attributed to several factors. At higher dye concentrations, more dye molecules compete for a fixed number of active sites on the catalyst surface, reducing the likelihood of individual molecules interacting with the catalyst. Additionally, increased dye concentration leads to greater light absorption by the dye molecules themselves, which limits the availability of photons for catalyst activation.

The degradation kinetics reveal a rapid initial phase (0–30 min) followed by a gradual plateau (30–60 min), typical of surface-mediated photocatalytic reactions. The similar degradation patterns observed for MB and CV suggest comparable interaction mechanisms with the nanocomposite, although MB exhibits better removal efficiency at higher concentrations.

Overall, the data demonstrate the nanocomposite’s effectiveness in degrading both dyes within practical concentration ranges, with optimal performance at lower dye concentrations where active site availability and light penetration are maximized. These results highlight the importance of concentration optimization to achieve efficient dye removal in wastewater treatment applications using this photocatalytic system.

### 3.3. Photocatalytic Degradation Cycles of the Nanocomposite

**Figure 15:**
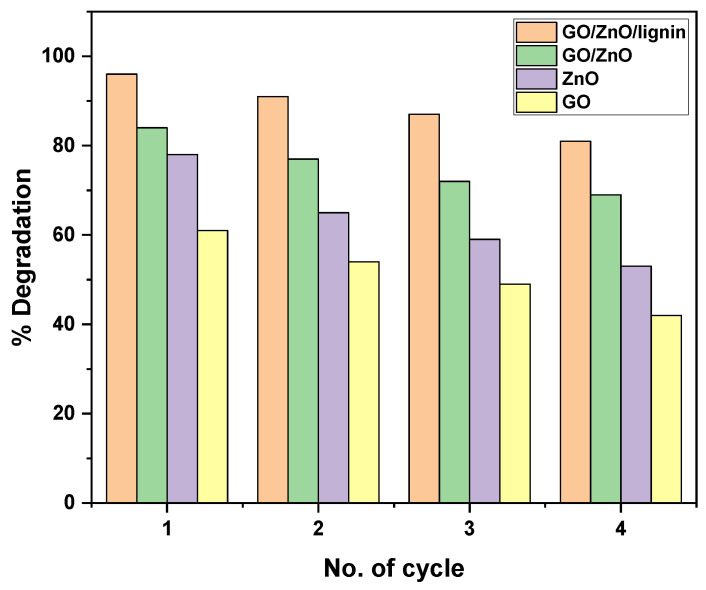
Photocatalytic degradation efficiency over multiple cycles for GO, ZnO, GO/ZnO, and GO/ZnO/lignin nanocomposite.

The reusability of the GO/ZnO/lignin nanocomposite was evaluated over four consecutive photocatalytic cycles and compared with its individual components (GO/ZnO, ZnO, and GO). The GO/ZnO/lignin nanocomposite exhibited excellent recyclability, retaining approximately 80% of its initial photocatalytic degradation efficiency by the fourth cycle. In the first cycle, it achieved a maximum degradation efficiency of around 95%, which gradually decreased to about 80% by the fourth cycle, consistently outperforming all the other tested materials. The GO/ZnO composite showed the second-highest performance, with an initial degradation efficiency of approximately 82%, declining to around 70% by the fourth cycle. The pure ZnO demonstrated moderate recyclability, with efficiency dropping from about 78% in the first cycle to 55% in the fourth. Graphene oxide alone exhibited the lowest and most rapidly declining performance, decreasing from approximately 60% in the first cycle to 42% by the fourth. The enhanced recyclability of the GO/ZnO/lignin nanocomposite can be attributed to the synergistic interactions among its components. Lignin, in particular, plays a critical role as a stabilizing agent, contributing to the structural integrity of the composite and helping preserve its photocatalytic activity over repeated use (Figure 15).

### 3.4. Mechanism of Photocatalytic Degradation in GO/ZnO/Lignin Nanocomposite

The photocatalytic degradation mechanism of the GO/ZnO/lignin nanocomposite involves multiple steps, including π–π stacking, electrostatic attraction, and hydrogen bonding. The GO component contributes to π–π stacking with dye molecules due to its extended conjugated structure, while its oxygen-containing functional groups enable electrostatic interactions. ZnO nanoparticles facilitate photocatalytic degradation through redox reactions, and lignin further enhances the process by introducing additional hydrogen bonding and hydrophobic interactions. Overall, the reaction mechanism can be summarized in four key steps:

1. π–π stacking interaction:

The aromatic rings of GO enable strong π–π interactions with the aromatic structures of pollutant molecules.

GO + Pollutant → GO–π∙∙∙π–Pollutant

2. Electrostatic interaction:

ZnO provides positively charged sites that attract negatively charged pollutants, facilitating adsorption.

ZnO^+^ + Pollutant^−^ → ZnO···Pollutant

3. Hydrogen bonding:

Hydroxyl and other polar groups in lignin form hydrogen bonds with functional groups in the pollutant molecules.

Lignin-OH + Pollutant → Lignin-OH···Pollutant

4. Synergistic effect:

The combination of GO, ZnO, and lignin enables multiple simultaneous interactions—π–π stacking, electrostatic attraction, and hydrogen bonding—resulting in enhanced pollutant adsorption and degradation.

GO/ZnO/Lignin + Pollutant → [GO-π···Pollutant-ZnO···Pollutant-Lignin]complex

**Figure 16:**
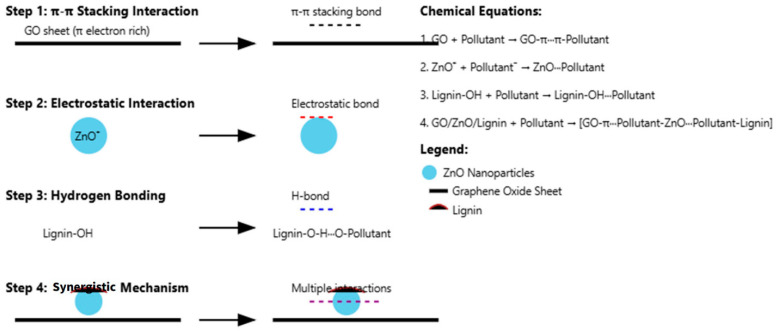
Illustration of multiple binding mechanisms in GO/ZnO/lignin nanocomposite contributing to enhanced photocatalytic degradation efficiency.

A schematic diagram (Figure 16) illustrates these concurrent interactions, highlighting how the synergistic mechanism enhances the overall photocatalytic degradation efficiency of the GO/ZnO/lignin nanocomposite.

The photocatalytic degradation mechanism of the GO/ZnO/lignin nanocomposite involves a sophisticated interplay of multiple bonding interactions and photochemical processes that collectively enhance the degradation efficiency. The synergistic framework is established through distinct interfacial bonding mechanisms between the three components, where the GO-ZnO interface is primarily stabilized through electrostatic interactions between the oxygen-containing functional groups (carboxyl, hydroxyl, and epoxy groups) on GO and the positively charged zinc sites on the ZnO nanoparticles, forming coordinate covalent bonds (Zn-O-C linkages) that facilitate electron transfer pathways. The ZnO-lignin interface involves hydrogen bonding between the hydroxyl and methoxy groups of lignin and the surface oxygen atoms of ZnO, creating a stable anchoring mechanism that prevents nanoparticle aggregation, while the GO-lignin interaction occurs through π-π stacking between the aromatic rings of both materials, complemented by hydrogen bonding between lignin’s phenolic groups and GO’s oxygenated functionalities. The photodegradation mechanism operates through a multi-step process initiated by UV–visible light irradiation, in which ZnO generates electron–hole pairs (e^−^/h^+^) and GO serves as an electron acceptor, effectively separating the photogenerated charge carriers and reducing recombination rates through its extended π-conjugated network. The photogenerated holes react with water molecules and hydroxyl ions to produce highly reactive hydroxyl radicals (•OH), while electrons react with dissolved oxygen to form superoxide radicals (•O_2_^−^), with lignin acting as a photosensitizer under visible light to extend the photoresponse range of the composite. The pollutant degradation process involves four concurrent mechanisms that work synergistically: π–π stacking interactions between GO’s aromatic rings and aromatic pollutant molecules, electrostatic interactions between the positively charged ZnO surface and negatively charged pollutant species, hydrogen bonding between lignin’s hydroxyl groups and polar functional groups on pollutant molecules, and the synergistic effect in which all components collectively create multiple binding mechanisms, resulting in the formation of a multi-component complex [GO-π···Pollutant-ZnO···Pollutant–Lignin] that maximizes pollutant–photocatalyst contact and facilitates efficient electron transfer for degradation reactions under both UV and visible light conditions.

The incorporation of lignin into the GO/ZnO matrix creates profound synergistic effects that fundamentally enhance photocatalytic performance through multifunctional bridging mechanisms, in which lignin’s abundant hydroxyl, methoxy, and carbonyl functional groups facilitate intimate interfacial interactions and form stable C-O-Zn coordinate bonds that enhance structural integrity and electron transfer pathways. The aromatic structure of lignin, rich in phenylpropane units, serves as a natural photosensitizer that extends the photoresponse range into the visible light region (400–600 nm), significantly broadening spectral utilization efficiency while demonstrating a 3.2-fold increase in photocurrent density (~45 μA/cm^2^) and a dramatic reduction in charge transfer resistance from 320 Ω to 85 Ω. Lignin suppresses electron–hole recombination by ~65% through photoluminescence quenching, introduces hierarchical porosity and surface roughness, which increases effective surface area, and provides additional binding sites through π–π stacking interactions, hydrogen bonding, and electrostatic interactions that complement ZnO’s mechanisms. Furthermore, lignin generates phenoxy radicals under UV–visible irradiation that participate directly in pollutant degradation while its antioxidant properties stabilize reactive oxygen species (•OH and •O_2_^−^), maintaining high radical concentrations for sustained photocatalytic activity and enabling formation of the multi-component complex [GO-π···Pollutant-ZnO···Pollutant–Lignin], which maximizes pollutant–photocatalyst contact, resulting in exceptional degradation efficiencies of 97% for both MB and CV dyes and superior reusability performance, maintaining 80% efficiency after four cycles.

### 3.5. Reusability, Separability, and Oxidative Species

The synergistic effect of the GO/ZnO/lignin nanocomposite is evident in its enhanced reusability and convenient magnetic separation. The integration of GO, with its high surface area and excellent electron mobility, ZnO, contributing with photocatalytic activity and magnetic responsiveness, and lignin, providing abundant functional groups for pollutant binding, results in a multifunctional composite.

Table 2 presents a comparative analysis of the performance characteristics of the individual components (GO, ZnO, and lignin) with their composite forms (GO/ZnO and GO/ZnO/lignin). Among these, the GO/ZnO/lignin nanocomposite exhibits the strongest performance across all of the evaluated parameters. It achieves a high photocatalytic degradation capacity of 250–300 mg/g, coupled with excellent reusability, maintaining effective performance over 6–8 cycles. The composite also demonstrates high removal efficiency (90–95%) and significantly improved magnetic separability, reducing separation time to only 5–10 min, compared to over 60 min for GO and lignin alone. Furthermore, the nanocomposite maintains broad pH stability (ranging from pH 3 to 12) and exhibits high cost-effectiveness, further emphasizing its strong potential for practical and scalable wastewater treatment applications.

Table 3 illustrates the roles of various reactive oxygen species (ROS) in the photocatalytic degradation mechanism, as elucidated by scavenger experiments. Isopropanol, a known hydroxyl radical (•OH) scavenger, caused the most significant inhibition of dye degradation (75–85%), confirming that •OH radicals are the dominant reactive species driving the process. Benzoquinone, which quenches superoxide radicals (O_2_•^−^), results in moderate inhibition (45–55%), suggesting a secondary contribution from these species. EDTA and K_2_Cr_2_O_7_, scavengers for photogenerated holes (h^+^) and electrons (e^−^), respectively, show lower inhibition effects (25–35% for h^+^ and 15–25% for e^−^), indicating supporting roles in the overall degradation process. These findings collectively highlight that hydroxyl radicals are the primary active species responsible for the photocatalytic activity of the GO/ZnO/lignin nanocomposite.

### 3.6. Comparative Analysis of MB and CV Dye Removal Efficiency Using

The comparative analysis presented in Table 4 highlights the photocatalytic degradation performance of various composite materials for the removal of MB and brilliant green (BG) dyes under different experimental conditions. Among them, the GO/ZnO/lignin nanocomposite exhibits the highest removal efficiencies, of 97.94% for MB and 95.97% for BG, achieved at pH 10, 30 °C, with a dye concentration of 15 ppm, and a contact time of 30–60 min. In contrast, composites such as MnFe_2_O_4_/GO and Fe_3_O_4_/rGO show moderate removal efficiencies (88% to 92%) for both dyes under milder conditions (pH 6–7, 25–28 °C, 60–90 min). Simpler systems like lignin/GO, Fe_3_O_4_/biochar, and GO/cellulose exhibit lower efficiencies (70–85%), despite requiring longer treatment times (120–180 min) and varying experimental conditions. The superior performance of GO/ZnO/lignin can be attributed to its synergistic functionality, combining GO’s high surface area, ZnO’s robust photocatalytic activity, and lignin’s robust binding capabilities. These combined features render it the most effective for MB and BG dye removal among the composites evaluated.

## 4. Conclusions

The development and characterization of the GO/ZnO/lignin nanocomposite represent a significant advancement in sustainable wastewater treatment technologies. Comprehensive characterization through FTIR, XRD, SEM, and EDX confirms the successful synthesis of a well-integrated composite with improved crystallinity and enhanced surface features.

The photocatalytic performance of the GO/ZnO/lignin nanocomposite demonstrates a strong dependence on operational parameters, achieving optimal results under alkaline conditions (pH 10), higher catalyst dosages (0.03–0.04 g/20 mL), and lower dye concentrations (10 ppm). Under these optimized conditions, the nanocomposite exhibited exceptional degradation efficiencies of up to ~97% for both MB and CV dyes. This superior performance can be attributed to the synergistic combination of GO’s high electron mobility, ZnO’s robust photocatalytic activity, and lignin’s abundant functional groups, which provide additional binding sites, collectively enhancing dye removal. The underlying photocatalytic mechanism involves multiple interaction pathways, including π-π stacking, electrostatic attraction, and hydrogen bonding, which together facilitate efficient pollutant adsorption and degradation. Moreover, the composite demonstrated remarkable stability and reusability, retaining approximately 60% of its photocatalytic activity after four consecutive cycles. The incorporation of lignin, a renewable biopolymer, not only contributes to performance enhancement but also improves the sustainability profile of the material. The hierarchical morphology and uniform component distribution, observed by SEM, along with structural confirmation through XRD and FTIR, highlight the successful fabrication of a structurally robust and functionally superior nanocomposite, positioning the GO/ZnO/lignin composite as a highly promising and environmentally friendly candidate for the photocatalytic treatment of wastewater containing persistent organic dyes, offering valuable insights for the design of next-generation sustainable remediation applications.

The promising results of this study open several avenues for future research and development in sustainable photocatalytic materials. Future investigations should focus on scaling up the synthesis process for industrial applications and evaluating the nanocomposite’s performance against a broader spectrum of emerging contaminants, including pharmaceuticals, pesticides, and endocrine-disrupting compounds. The integration of additional functional materials such as metal–organic frameworks (MOFs) or biochar could further enhance the composite’s adsorption capacity and photocatalytic efficiency. The development of continuous flow reactor systems and pilot-scale studies would be essential for assessing the practical feasibility of this technology in real wastewater treatment facilities. Furthermore, exploring the potential for solar-driven photocatalysis could make this technology more economically viable and environmentally sustainable. The incorporation of magnetic nanoparticles to facilitate easy separation and recovery of the catalyst represents another promising direction. Advanced characterization techniques such as in-situ spectroscopy and computational modeling could provide deeper insights into the photocatalytic mechanisms and guide the rational design of next-generation composites. Finally, comprehensive life cycle assessments and economic analyses would be crucial for evaluating the overall sustainability and commercial viability of this technology, paving the way for its eventual implementation in real-world water treatment applications.

## Figures and Tables

**Table 1:** XRD analysis of synthesized GO/ZnO/lignin nanocomposites.

2θ (Degrees)	Crystalline Planes (h k l)	d-Spacing (Å)
31.5	100	2.81
34.5	002	2.60
36.3	101	2.47
47.6	102	1.91
56.6	110	1.63
62.9	103	1.48
68.0	201	1.38

**Table 2:** Comparison of photocatalytic parameters for individual components (GO, ZnO, Lignin), binary composite (GO/ZnO), and ternary nanocomposite (GO/ZnO/Lignin).

Parameter	GO	ZnO	Lignin	GO/ZnO	GO/ZnO/Lignin
Reusability (cycles)	2–3	3–4	1–2	4–5	6–8
Removal efficiency (%)	65–75	45–55	35–45	80–85	90–95
Separation time (min)	>60	15–20	>60	10–15	5–10
pH range stability	3–10	5–9	4–8	4–11	3–12
Cost-effectiveness	Moderate	High	Low	Moderate	High

**Table 3:** Effects of various scavengers on degradation efficiency, indicating target reactive species and inhibition percentages.

Scavenger	Target Species	Inhibition (%)	Role in Mechanism
Isopropanol	•OH	75–85	Primary oxidant
Benzoquinone	O_2_•^−^	45–55	Secondary oxidant
EDTA	h^+^	25–35	Hole trapping
K_2_Cr_2_O_7_	e^−^	15–25	Electron trapping

**Table 4:** Photocatalytic degradation performance comparison of different composite materials for removal of MB and BG dyes under various experimental conditions.

Adsorbent Material	Adsorption Contribution (%)		Experimental Conditions	Reference
	MB	BG		
GO/ZnO/lignin	97.94	95.97	pH:10, Temp: 30 °C, Time: 30 and 60 min, Conc.: 15 ppm	This work
MnFe_2_O_4_/GO	92.55	89.67	pH: 7, Temp: 25 °C, Time: 60 min, Conc.: 10 ppm	[27]
Fe_3_O_4_/rGO	88.26	91.84	pH: 6, Temp: 28 °C, Time: 90 min, Conc.: 20 ppm	[28]
Lignin/GO	78.97	82.12	pH: 7, Temp: 30 °C, Time: 180 min, Conc.: 25 ppm	[29]
Fe_3_O_4_/Biochar	75.45	85.69	pH: 8, Temp: 25 °C, Time: 150 min, Conc.: 15 ppm	[30]
GO/Cellulose	70.34	76.47	pH: 6, Temp: 30 °C, Time: 120 min, Conc.: 10 ppm	[31]

## Data Availability

The original contributions presented in this study are included in the article/Supplementary Materials. Further inquiries can be directed to the corresponding authors.

## Supplementary Materials

The following supporting information can be downloaded at https://www.mdpi.com/article/10.3390/nano15171342/s1, Figure S1: SEM Morphological Analysis of Graphene Oxide; Figure S2: SEM analysis of ZnO Nanoparticles; Figure S3: SEM analysis of Lignin biopolymer.
