# Supplementary material for: Highly Efficient Graphene Oxide/Zinc Oxide/Lignin Catalyst for Photocatalytic Degradation of Methylene Blue and Gentian Violet
Source: Nanomaterials (Basel). 2025 Sep 1;15(17):1342. doi: 10.3390/nano15171342 (PMC12430695; doi:10.3390/nano15171342)
Supplement: Supplementary file 1 [file nanomaterials-15-01342-s001.zip › nanomaterials-3742088-supplementary.pdf]

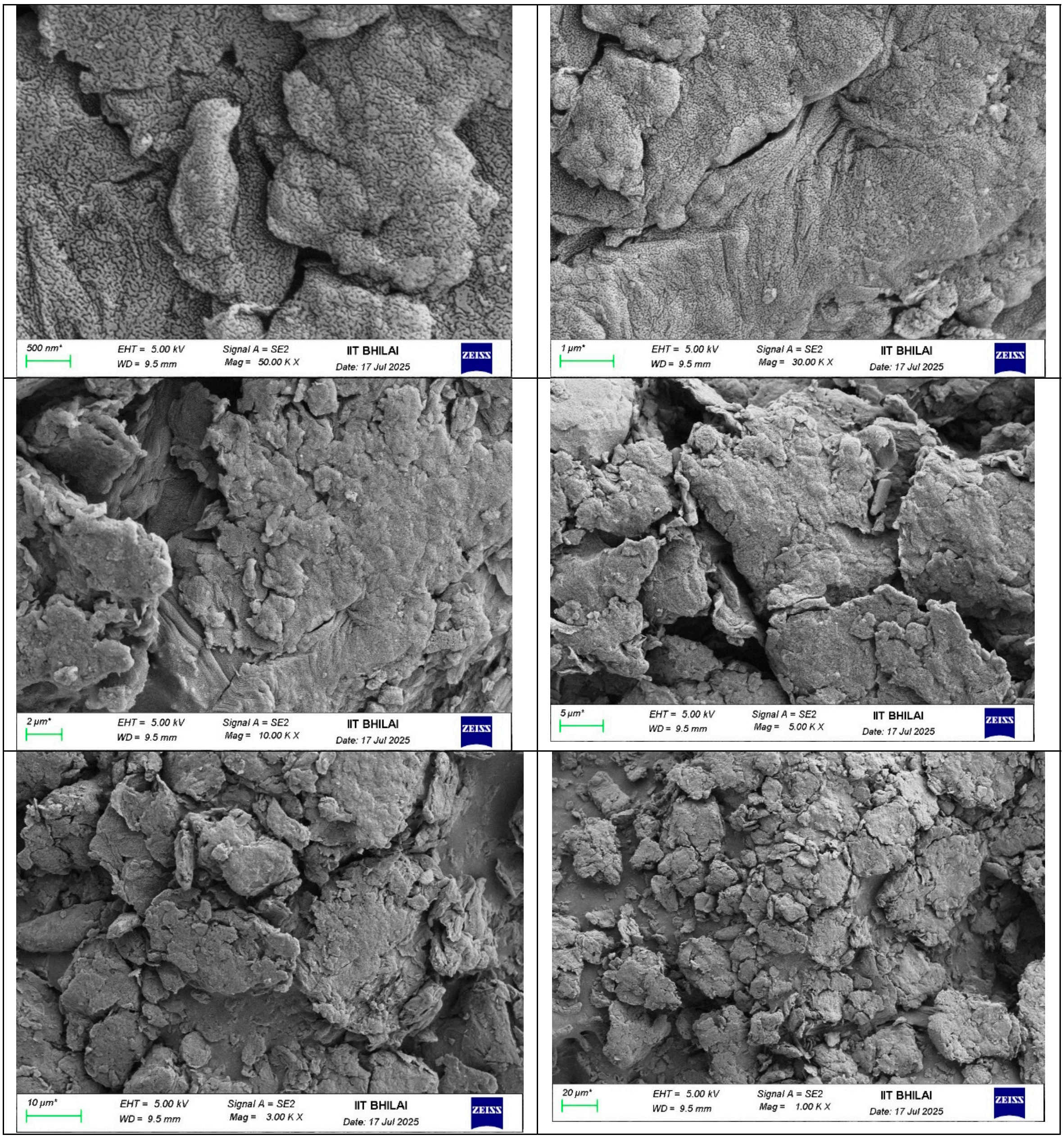

Figure S1 SEM Morphological Analysis of Graphene Oxide

The SEM images of graphene oxide reveal the characteristic two-dimensional sheet structure with distinctive wrinkled and folded morphology resembling crumpled paper. These ultra-thin sheets, typically ranging from single to few layers thick, exhibit excellent transparency under electron beam exposure, confirming their atomic-level thinness. The wrinkles and folds are inherent structural features formed during the oxidation and exfoliation process of graphite, not defects, and actually increase the surface area compared to perfectly flat sheets. The smooth surface morphology at higher magnifications demonstrates the high-quality layered structure, while overlapping regions where multiple sheets stack together create darker areas due to increased electron scattering. The extensive sheet-like structures provide maximum surface exposure, making graphene oxide particularly valuable for applications requiring high surface

area such as energy storage, catalysis, and composite materials. The clearly visible edges show the extent of individual sheets and their layered arrangement, highlighting the material's potential for various technological applications.

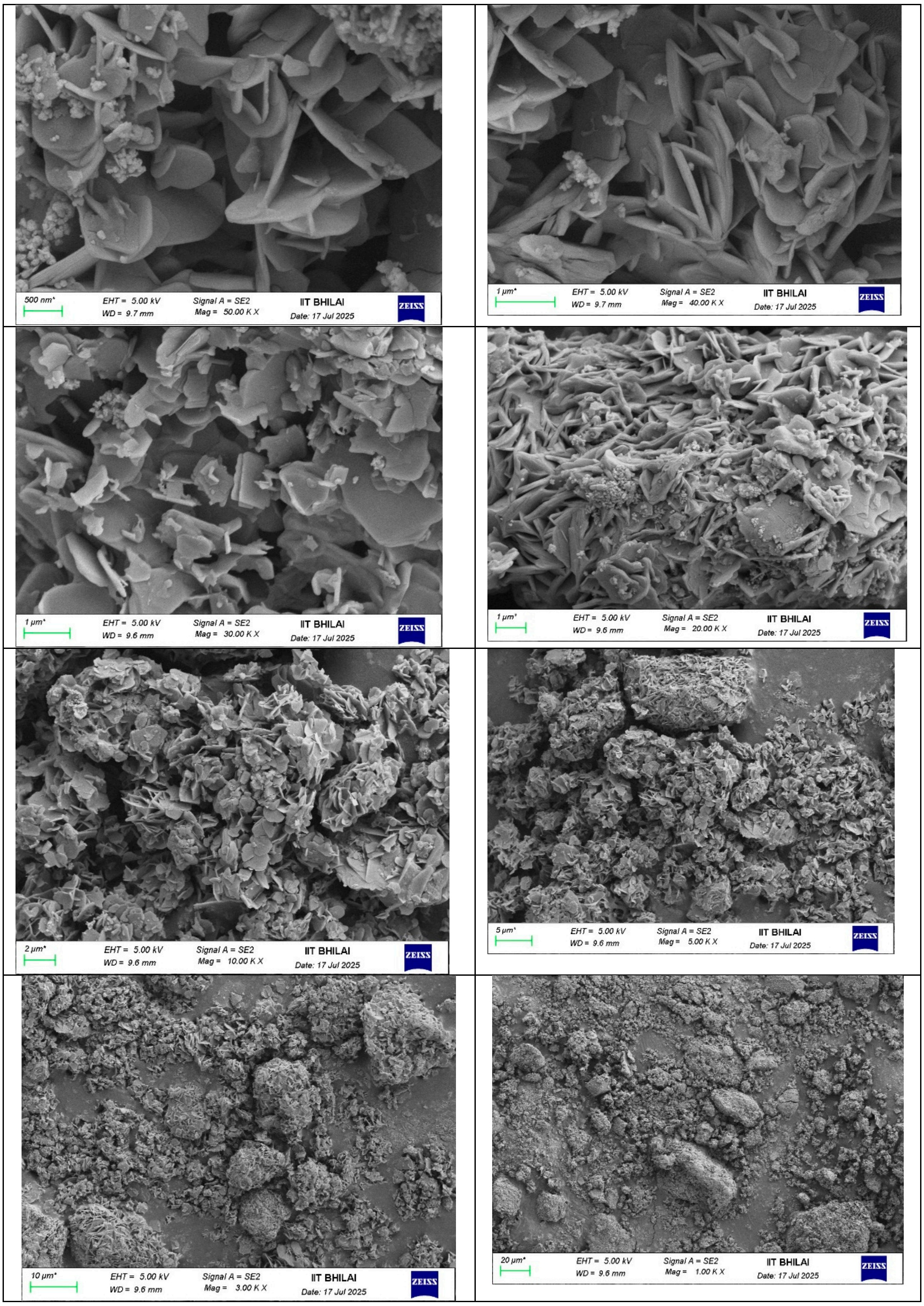

Figure S2 SEM analysis of ZnO Nanoparticles

The SEM images of zinc oxide nanoparticles showcase highly crystalline structures with well-defined geometric shapes, typically exhibiting hexagonal morphologies characteristic of the

wurtzite crystal structure. The particles display excellent crystallinity with clear faceted surfaces and relatively uniform size distribution, indicating controlled synthesis conditions. The smooth, well-defined surface features are crucial for understanding ZnO's photocatalytic, antimicrobial, and electronic properties, as these are largely surface-dependent phenomena. Some images may show particle agglomeration due to high surface energy, yet individual particles maintain their crystalline structure even when clustered. The morphology reflects the synthesis method effectiveness, with different techniques yielding varying particle shapes from spherical to rod-like structures. The uniform particle distribution and crystalline quality make ZnO nanoparticles suitable for applications in UV protection, photocatalysis, sensors, and antimicrobial treatments. The high surface area and well-defined crystal facets contribute to enhanced reactivity and functionality in various technological applications.

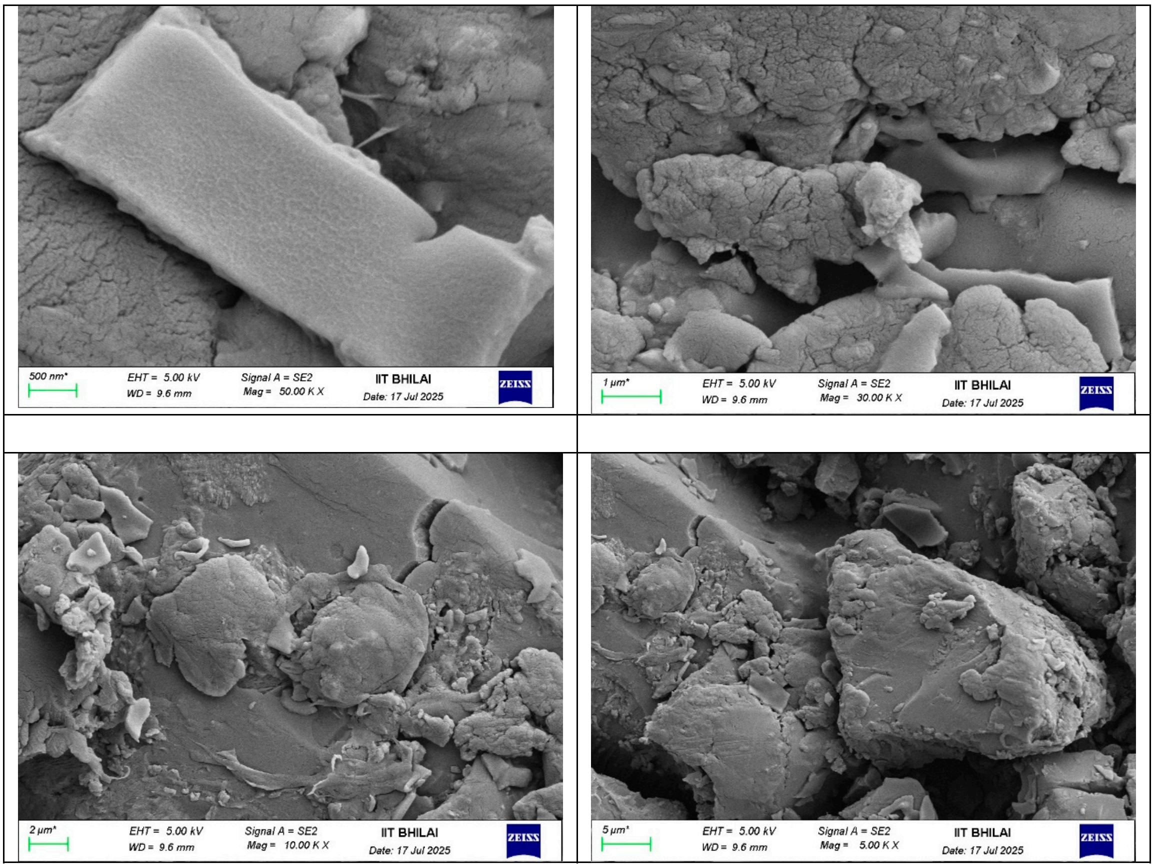

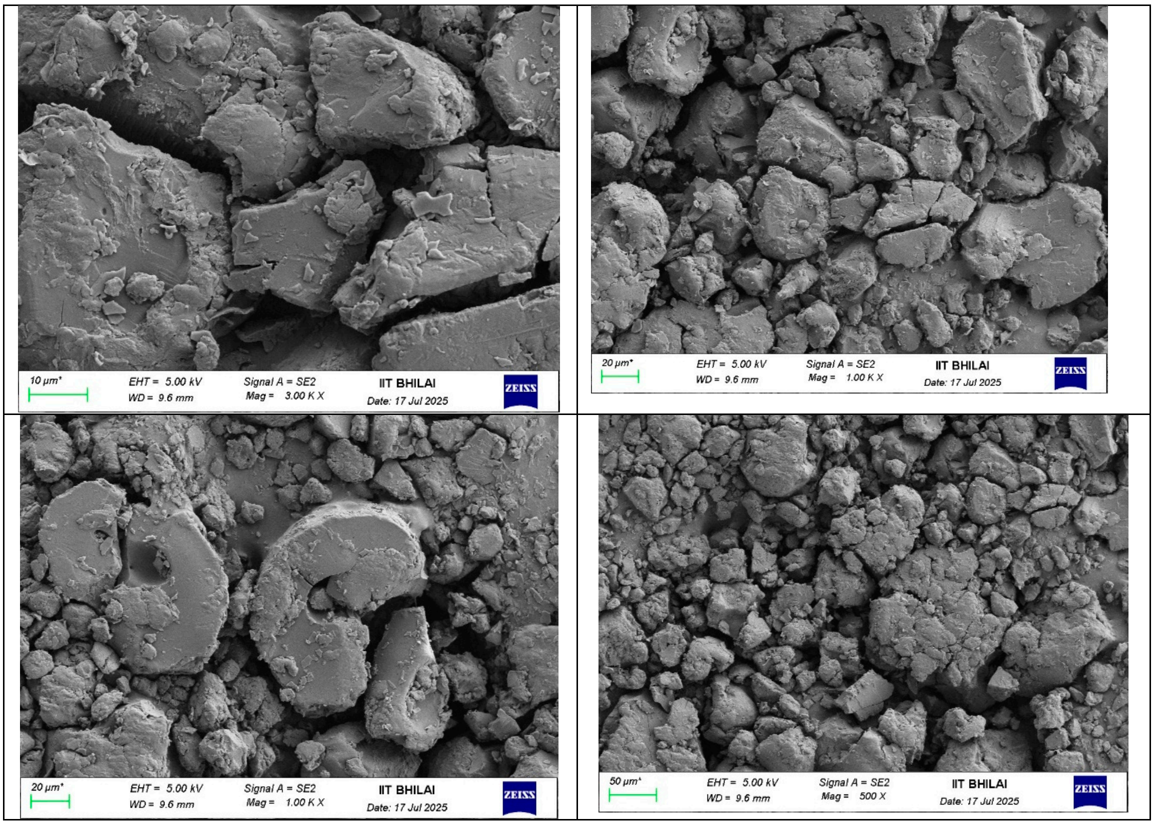

Figure S3 SEM analysis of Lignin biopolymer

The SEM images of lignin display the complex, amorphous nature of this biopolymer with highly irregular, heterogeneous morphology reflecting its natural polymeric network structure. Unlike synthetic materials, lignin exhibits significant structural heterogeneity due to its biological origin, showing variations in density, porosity, and surface features across different sample regions. The characteristic rough, porous surface texture results from lignin's complex molecular structure consisting of randomly cross-linked aromatic units, appearing fibrous or globular depending on source material and extraction method. The irregular surface morphology provides numerous sites for chemical modification, making lignin valuable for polymer composites, adhesives, and renewable alternatives to petroleum-based materials. The processing-induced features visible in the images reflect different extraction techniques (kraft, organosolv, enzymatic) that produce lignin with varying structural characteristics. The porous structure and complex morphology make lignin suitable for filtration, separation applications, and as a sustainable material in various industrial processes. The heterogeneous nature, while challenging for standardization, offers multiple functional sites that enhance its versatility in green chemistry applications.
